# Quantitative estimation of track segment yields of water radiolysis species under heavy ions around Bragg peak energies using Geant4-DNA

**DOI:** 10.1038/s41598-021-81215-6

**Published:** 2021-01-15

**Authors:** Kentaro Baba, Tamon Kusumoto, Shogo Okada, Ryo Ogawara, Satoshi Kodaira, Quentin Raffy, Rémi Barillon, Nicolas Ludwig, Catherine Galindo, Philippe Peaupardin, Masayori Ishikawa

**Affiliations:** 1 Graduate School of Biomedical Science and Engineering, Hokkaido University, Kita-15 Nishi-7, Kita-ku, Sapporo, Hokkaido 060-8638 Japan; 2 National Institutes for Quantum and Radiological Science and Technology, 4-9-1 Anagawa, Inage-ku, Chiba, 263-8555 Japan; 3 High Energy Accelerator Research Organization (KEK), 1-1, Oho, Tsukuba, Ibaraki 305-0801 Japan; 4 Advanced Research Center for Beam Science, Institute for Chemical Research, Kyoto University, Gokasho, Uji, Kyoto 611-0011 Japan; 5 Institute Pluridisiplinaire Hubert Curien, UMR 7178, CNRS, 23 rue du Loess, 67037 Strasbourg, France; 6 Faculty of Health Sciences, Hokkaido University, Kita-12 Nishi-5, Kita-ku, Sapporo, Hokkaido 060-0812 Japan

**Keywords:** Particle physics, Information theory and computation

## Abstract

We evaluate the track segment yield G′ of typical water radiolysis products (e_aq_^−^, ^**·**^OH and H_2_O_2_) under heavy ions (He, C and Fe ions) using a Monte Carlo simulation code in the Geant4-DNA. Furthermore, we reproduce experimental results of ^**·**^OH of He and C ions around the Bragg peak energies (< 6 MeV/u). In the relatively high energy region (e.g., > 10 MeV/u), the simulation results using Geant4-DNA have agreed with experimental results. However, the G-values of water radiolysis species have not been properly evaluated around the Bragg peak energies, at which high ionizing density can be expected. Around the Bragg peak energy, dense continuous secondary products are generated, so that it is necessary to simulate the radical–radical reaction more accurately. To do so, we added the role of secondary products formed by irradiation. Consequently, our simulation results are in good agreement with experimental results and previous simulations not only in the high-energy region but also around the Bragg peak. Several future issues are also discussed regarding the roles of fragmentation and multi-ionization to realize more realistic simulations.

## Introduction

Cancer patients can nowadays select several modalities in radiotherapy such as conventional X-ray therapy and particle therapies (e.g., proton therapy, heavy ion therapy and boron neutron capture therapy). The number of patients receiving particle therapy for cancer (mainly proton or C-ion beams) is increasing annually^1^. There are two major advantages to particle therapy. The first is having a higher quality of life (QOL) compared to other modalities, such as surgical operation and/or chemotherapy. The second is high effectiveness for deep-seated hypoxic tumors. For particle therapies, high ionization density can be expected at the tumor site due to the Bragg peak^2^, around which a large amount of energies of incoming ions is deposited. The accuracy of dose calculation around the Bragg peak is thus very important for treatment planning. Monte Carlo simulations have recently been adopted as a treatment planning software application for dose calculations. Judgment for patient-specific quality assurance is performed based on the calculation result using a Monte Carlo simulation. For example, PTSim (Particle Therapy Simulation Framework) is a Geant4 based Monte Carlo simulation toolkit for particle therapy, that some institutes use clinically in Japan^3^. Furthermore, the radiation therapy program based on Geant4, named GATE, is often used in European countries^4^. Using Monte Carlo simulations, we can accurately calculate dose distributions even in complex geometries, for instance, the human body, only when truly precise physics processes are installed in the computer code. While primary energy transfer induced by ionizing radiation in media occurring in the extremely fast stage (typically 10^–18^ s)^5^ can be accurately calculated using a Monte Carlo simulation toolkit, it is tough to simulate the following secondary reactions in the “physicochemical phase” (from 10^–15^ to 10^–12^ s) and the “chemical phase” (from 10^–12^ to 10^–6^ s). For example, in the PTSim, only energy deposition is taken into account, meaning that DNA damage induced by water radiolysis species in the chemical phase is not considered. Water radiolysis species are abundantly formed along ion tracks. To consider roles played by water radiolysis species, we have to follow the reaction occurring with each water radiolysis product. However, it takes long time for computation. In the “physicochemical phase” for water radiolysis, initial products are reorganized into modified species (e.g., stable molecules and water-free radicals) and thermal equilibrium in the bulk media is established. Reaction processes are then extended up to neutralization of the media in the “chemical phase”. During the "physicochemical phase" and the "chemical phase", water radiolysis products can damage DNA molecules. The correlation between yields of water radiolysis products and those of DNA molecules damaged should therefore be assessed to elucidate the contribution of indirect action.

Many researchers have experimentally evaluated radiation chemical yields (G-values), the number of entities formed or destroyed by unit energy (conventionally 100 eV), of ^**·**^OH, hydrated electrons (e^−^_aq_) and hydrogen peroxide (H_2_O_2_), all of them are typical water radiolysis products under ionizing radiation^6–10^, using radical scavengers (e.g., coumarin-3-carboxylic acid (C3CA) and phenol). Furthermore, the correlation between generated yields of ^**·**^OH, which efficiently react with DNA molecules, and those of radiolysis product of an amino acid disassembled in solution is being quantitatively clarified^11^. As is well known, the effectiveness of indirect action increases with the increasing of the energy of incoming ions, while the linear energy transfer (LET) decreases^10,12^. Around the Bragg peak of C ions, the contribution of indirect action is up to 50% for cell killing under humidified air with 5% CO_2_ at 37 ℃^13^. Chemical reactions by water radiolysis products should therefore be taken into account to properly understand the biological effectiveness of cancer treatment with radiation^14^.

In parallel with experiments, the LET dependence of G-values of typical water radiolysis products in the high energy region (> 100 MeV/u) was evaluated using Geant4-DNA^15,16^ The simulation results were in agreement with experimental results. However, G-values around Bragg peak energies have not been successfully simulated. Even at the Bragg peak energy region of C ions, the contribution of indirect action for biological effects is still large (~ 50%). We are now trying to establish a platform that can estimate the therapeutic effects and evaluate biological effects by simulations with reference to the amount of ^**·**^OH obtained experimentally. In this work, we simulate G-values of ^**·**^OH, e_aq_^−^ and H_2_O_2_ under heavy ions (He, C and Fe ions) up to 10^–6^ s using Geant4-DNA. The obtained simulation results are compared to the experimental results around Bragg peak energies and previously obtained ones in the high energy region.

## Materials and methods

### Experiments

Previously, yields of water radiolysis products (^**·**^OH, e_aq_^−^ and H_2_O_2_) under ion irradiation were evaluated. In this section, we reproduce the derivation process of the yields reported in the previous study^11^.

G-values of ^**·**^OH have been measured using radical scavengers [e.g., phenol and coumarin-3-carboxilic (C3CA)] in solution^9–11,17^. C3CA (purity 0.98%; Fujifilm/Wako Pure Chemical Industries Ltd., Osaka, Japan) with phosphate buffer was prepared in ultrapure water (Milli-Qt Advantage; Merck & Co., Kenilworth, NJ) at 66 mM, pH = 6.8^17^. From these measurements, the number of ^**·**^OH formed per ion track (N) can be computed.

1 $${\text{N}} = \frac{{{\text{G}}\left( {^{ \cdot } {\text{OH}}} \right) \cdot {\text{E}}_{0} }}{100} = \frac{1}{100}\mathop \smallint \limits_{0}^{{{\text{E}}_{0} }} {\text{G}}^{\prime } \left( {^{ \cdot } {\text{OH}}} \right){\text{dE,}}$$

where E_0_ is the initial energy of incoming ions. When the experiments are done at several energies, N can be plotted as a function of the energy of the ions, and a resulting fitting curve can be derived. Then, we can determine the track segment yield G′, that is the yield for an infinitesimal energy loss, defined for one specific energy of ion:

2 $${\text{G}}^{\prime } \left( {^{ \cdot } {\text{OH}}} \right) = 100 \times \frac{{{\text{dN}}}}{{{\text{dE}}}}.$$

The so-called “scavenging capacity” of a scavenging reaction is expressed by the product of the rate constant of the reaction and the concentration of the radical scavenger. Its inverse represents the average scavenging time scale. So, by adjusting the concentration of probe, the time dependence of the G-value of ^**·**^OH can be reconstructed^6^. The detailed procedures for experiments to evaluate G-values of ^**·**^OH are described elsewhere^10,11,17^.

The aqueous solution containing 1 mM sodium nitrate (NaNO_3_) and 5 mM di-sodium hydrogen phosphonate (Na_2_HPO_3_) are used for the measurement of e_aq_^−^. e_aq_^−^ is scavenged by nitrate anion to produce NO_3_^2−**·**^, which immediately reacts with one of the surrounding water molecules, resulting in the production of nitrogen dioxide (NO_2_^**·**^). NO_2_^**·**^ then reacts with hydrogen phosphate anion (HPO_3_^2−^) to produce an anion (NO_2_^−^), which can easily be determined by applying Saltzman technique^18^ with molar extinction coefficient of 42,3000 M^−1^ cm^−1^ at 540 nm. Furthermore, Ghormley technique^19^ has been adopted to evaluate the yield of H_2_O_2_. H_2_O_2_ is very stable compared to other water radiolysis products but is destroyed by e_aq_^−^ due to intra-track reactions. To minimize the decomposition by intra-track reactions, 2.5 mM NaNO_3_ aqueous solution was used for measurements. The detailed procedures for experiments are described elsewhere^8^.

In this work, we re-plotted these experimental results. All experiments were done under neutral pH conditions at 25 ℃^7,8,10^.

### Simulations

A Monte Carlo simulation with Geant4-DNA was performed to calculate G-values of water radiolysis products^20–26^. We used the *G4EmDNAPhysics_option8* physics constructor with additional sub-excitation processes of vibrational excitation and molecular attachment for electrons installed in Geant4-DNA ver. 10.05.p01. The *G4EmDNAPhysics_option8* physics constructor covers the energy range from 0.5 MeV/u to 10^6^ MeV/u for heavy ions (He, C and Fe ions). Furthermore, *G4EmDNAChemistry* was used for the simulation in the chemical stage. The simulation geometry is a 10 × 10 × 10 mm^3^ air-free water cube. This is the size determined in consideration of the measurement system. When primary particles have deposited 10 keV of their energies into the water, the charged particle tracking simulation of the physical process was ceased and shifted to the chemical process. We aborted the event when total energy deposition of each event exceeded 10.1 keV in order to simulate large enough numbers of tracks in reasonable calculation times. That means that total energy deposition of each event was always between 10 and 10.1 keV. In Geant4-DNA, we can follow a particle-based approach for the simulation of water radiolysis^27^, where molecular species are modeled as point-like objects diffusing in a continuous liquid water medium. In this work, we follow the radical annihilation process from 1 ps to 1 μs.

When energies of incoming ions were high enough (e.g., 100 MeV, 25 MeV/u and 400 MeV/u for protons, He and C ions, respectively), they could easily go through a water cube of 10 mm thickness. If not, incoming ions are completely stopped in the water cube. In such cases, energies of incoming ions were rapidly lost, meaning that high ionizing density can be expected around the Bragg peak. This implied that water radiolysis products could be abundantly generated along ion tracks. So, considering the issue of calculation time, it is not easy to follow the chemical process around the Bragg peak energy using a CPU-based simulator. In this study, the issue was overcome by a split simulation. The details of the split simulation are discussed in the following section.

Substantial yields of secondary products generated by water radiolysis (e.g., O^**·**−^, O_2_, O_2_^**·**−^, HO_2_^**·**^, HO_2_^−^) have been previously evaluated^28–30^. In this study, we defined additional molecular species shown in Table 1, which are not implemented in Geant4-DNA ver. 10.05.p01, considering the number of atoms, the number of occupied electrons, the electron occupancy (depending on the molecular composition of each molecule), the Van der Waals radius, mass, charge and diffusion coefficient produced in air-free water due to irradiation. The diffusion coefficients related to the newly defined products are listed in the right column of Table 1^31^. In the physical stage, the water molecules are excited or ionized, and initial radiolysis products are generated by dissociation in the physical–chemical stage^32,33^. The dissociation/association scheme leads to the formation of further radiolysis products such as H_2_, H^**·**^ and ^**·**^OH. The branching ratios used in this study are listed in Table 2. This table is recalled from a previous study^15^. In the chemical stage in Geant4-DNA, starting from 1 ps, the molecular species diffuse and can react together based on the diffusion coefficients and the chemical reaction rates. The list of reactions and reaction rate constants are shown in Table 3. The reaction rate constants were referred from a previous study^34,35^. The present simulation was done using *G4EmDNAChemistry* with the role of the newly defined molecules.

**Table 1:** Diffusion coefficients of the newly defined products from Frongillo et al.^31^.

Molecular species	Diffusion coefficient (10^–9^ m^2^ s^−1^)
O_2_	2.4
O_2_^**·**−^	1.75
HO_2_^**·**^	2.3
HO_2_^−^	1.4
O^**·**−^	2.0

**Table 2:** Dissociation schemes and branching ratios. This table is recalled from Shin et al.^15^.

Electronic state of water molecule	Dissociation channels	Probability
All single ionization states	H_3_O^+^ + ^**·**^OH	1
Excitation state: A1B1	^**·**^OH + H^**·**^	0.65
	H_2_O + ΔE	0.35
Excitation state: B1A1	H_3_O^+^ + ^**·**^OH + e_aq_^−^	0.55
	^**·**^OH + ^**·**^OH + H_2_	0.15
	H_2_O + ΔE	0.3
Excitation state	H_3_O^+^ + ^**·**^OH + e_aq_^−^	0.5
Rydberg, diffusion bands	H_2_O + ΔE	0.5
Dissociate attachment	^**·**^OH + OH^−^ + H_2_	1

**Table 3:** Reaction and reaction rate constants. These values are based on the data of Hatano et al.^34^. The reaction rate constants for first-order reactions of species O^・−^, HO_2_^−^ are based on the data reported by Plate^35^.

Reaction	Reaction rate (dm^3^ mol^−1^ s^−1^)	Reaction	Reaction rate (dm^3^ mol^−1^ s^−1^)
H^**·**^ + H^**·**^ → H_2_	5.03 × 10^9^	H_2_O_2_ + e_aq_^−^→ OH^−^ + ^**·**^OH	1.1 × 10^10^
H^**·**^ + ^**·**^OH → No product	1.55 × 10^10^	H_2_O_2_ + OH^−^ → HO_2_^−^	1.27 × 10^10^
H^**·**^ + H_2_O_2_ → ^**·**^OH	3.5 × 10^7^	H_2_O_2_ + O^**·**−^ → HO_2_^**·**^ + OH^−^	5.55 × 10^8^
H^**·**^ + e_aq_^−^→ H_2_ + OH^−^	2.5 × 10^10^	e_aq_^−^+ e_aq_^−^→ OH^−^ + OH^−^ + H_2_	5.0 × 10^9^
H^**·**^ + OH^−^ → e_aq_^−^	2.51 × 10^7^	e_aq_^−^+ H_3_O^+^ → H^**·**^	2.11 × 10^10^
H^**·**^ + O_2_ → HO_2_^**·**^	2.1 × 10^10^	e_aq_^−^+ O_2_^**·**−^ → H_2_O_2_ + 2OH^−^	1.3 × 10^10^
H^**·**^ + HO_2_^**·**^ → H_2_O_2_	1.0 × 10^10^	e_aq_^−^+ HO_2_^−^ → O^**·**−^ + OH^−^	3.51 × 10^9^
H^**·**^ + O_2_^**·**−^ → HO_2_^-^	1.0 × 10^10^	e_aq_^−^+ O_2_ → O_2_^**·**−^	1.74 × 10^10^
^**·**^OH + ^**·**^OH → H_2_O_2_	5.5 × 10^9^	e_aq_^−^+ HO_2_^**·**^ → HO_2_^−^	1.28 × 10^10^
^**·**^OH + H_2_O_2_ → HO_2_^**·**^	2.87 × 10^7^	H_3_O^+^ + O_2_^**·**−^→ HO_2_^**·**^	4.78 × 10^10^
^**·**^OH + H_2_ → H^**·**^	3.28 × 10^7^	H_3_O^+^ + OH^−^ → No product	1.13 × 10^11^
^**·**^OH + e_a_^−^→ OH^−^	2.95 × 10^10^	H_3_O^+^ + HO_2_^−^ → H_2_O_2_	5.0 × 10^10^
^**·**^OH + OH^−^ → O^**·**−^	6.3 × 10^9^	HO_2_^**·**^ + O_2_^**·**−^ → O_2_ + HO_2_^−^	9.7 × 10^7^
^**·**^OH + HO_2_^**·**^ → O_2_	7.9 × 10^9^	HO_2_^**·**^ + HO_2_^**·**^ → O_2_ + H_2_O_2_	8.3 × 10^5^
^**·**^OH + O_2_^**·**−^ → OH^−^ + O_2_	1.07 × 10^10^	O^・−^ + H_2_O → ^**·**^OH + OH^−^	1.36 × 10^6^ (s^−1^)
^**·**^OH + HO_2_^−^ → OH^−^ + HO_2_^**·**^	8.32 × 10^9^	HO_2_^−^ + H_2_O → H_2_O_2_ + OH^−^	1.36 × 10^6^ (s^−1^)
^**·**^OH + O^**·**−^ → HO_2_^−^	1.0 × 10^9^		

## Results and discussion

### Time dependence of G-value of water radiolysis products

**Figure 1:**
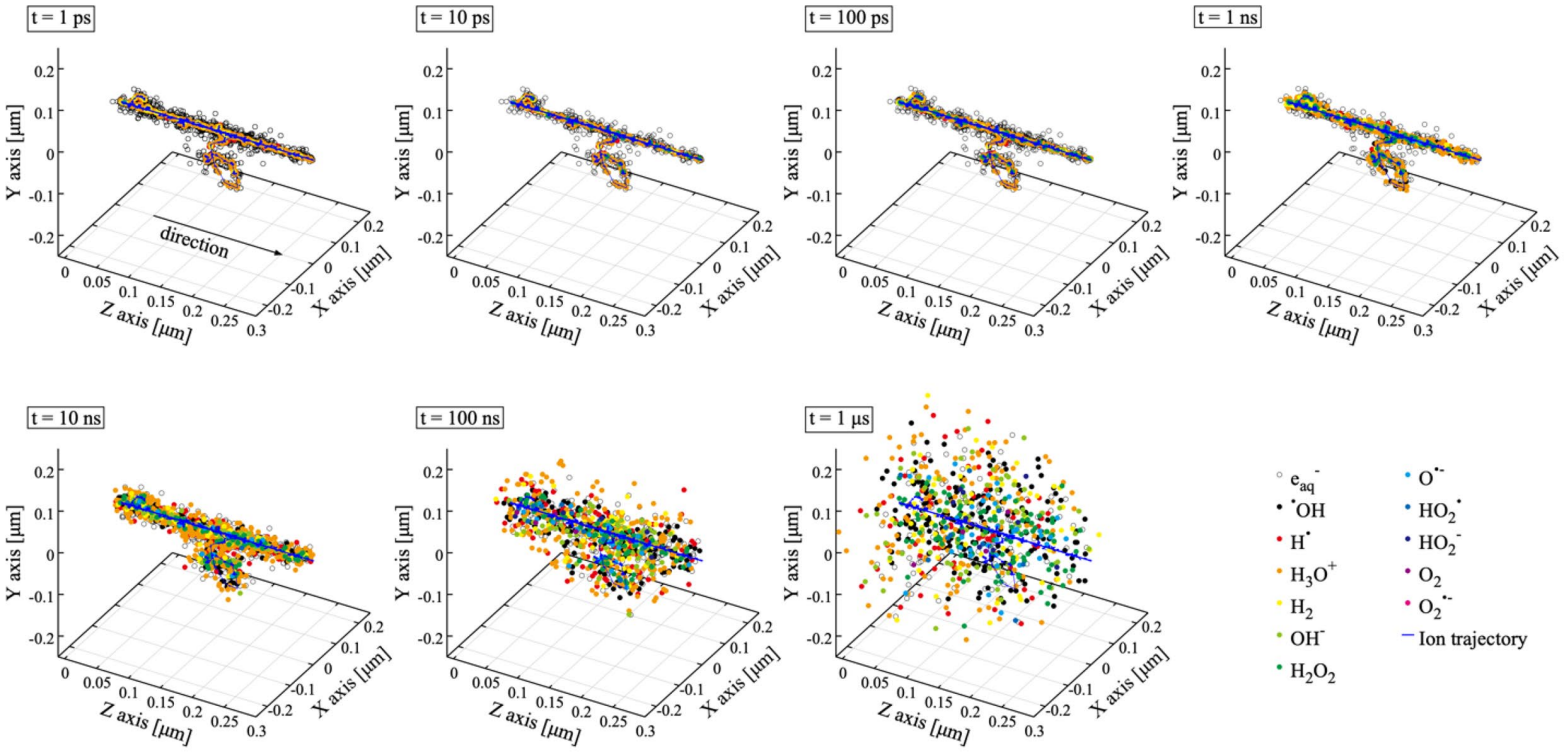
Chemical evolution of 400 MeV/u carbon ion track in water in the time 1 ps to 1 μs.

**Figure 2:**
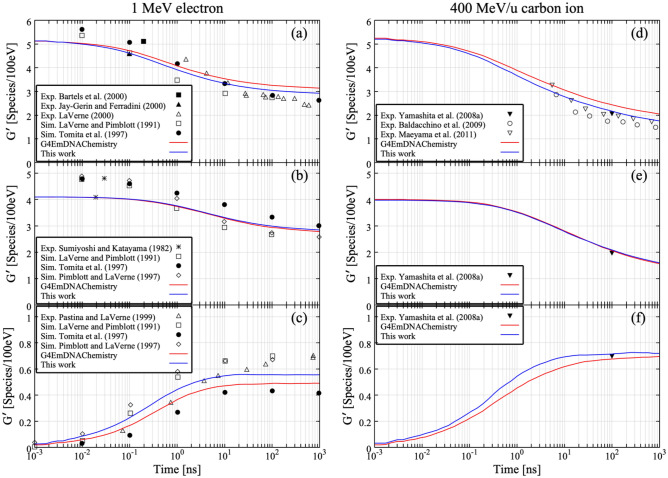
Time dependence of G-values of (**a**) ^**·**^OH, (**b**) e_aq_^−^ and (**c**) H_2_O_2_ for 1 MeV electrons, and (**d**) ^**·**^OH, (**e**) e_aq_^−^ and (**f**) H_2_O_2_ for 400 MeV/u carbon ions. The solid lines represent the results of Monte Carlo simulations. Sets of reference data^36–43^ were used for comparison with simulated time dependence of G-values.

Figure 1 illustrates the schematic view of diffusion of reactive species produced by water radiolysis from 1 ps to 1 μs after the irradiation of 400 MeV/u C ions. Water radiolysis products along the C ion path are denser than those along secondary electron trajectories. The high density of reactive species can be seen around the C ion trajectory. Reactive species then diffuse to distant locations from the ion path. Figure 2a–c show the time dependence of G-values of ^**·**^OH, e_aq_^−^ and H_2_O_2_ after irradiation of 1 MeV electrons. The number of launched ions/electrons was 1000 and the statistical error was around 5%. Our simulation results of reaction of water radiolysis products with newly defined molecules (e.g., O^**·**−^, O_2_, O_2_^**·**−^, HO_2_^**·**^, HO_2_^-^) are shown with a solid blue line and those without the reactions are shown with a solid red line (*G4EmDNAChemistry*). The previously obtained reference data are also plotted^36–43^. The simulation results are in good agreement with experimental values. The G-values of ^**·**^OH calculated using *G4EmDNAChemistry* are slightly higher than our simulation result, and closer to experimental data in several cases. This implies that the added reactions act significantly. However, there are variations in experimental results, so that it is difficult to argue if the present simulation with reactions of water radiolysis product with newly defined molecules is appropriate. In comparison with ^**·**^OH, no difference is observed between the two simulations for the G-values of e_aq_^−^. Furthermore, the G-value of H_2_O_2_ with the new reactions added is higher than that without the reactions. Indeed, H_2_O_2_ is not directly produced by water radiolysis, meaning that H_2_O_2_ is made by radical–radical reaction (e.g., ^**·**^OH + ^**·**^OH → H_2_O_2_). Since the G-value of H_2_O_2_ with the new reactions is higher than without them, the role played by secondary products seem to be important. Similar results are observed with 400 MeV/u C ions as shown in Fig. 2d–f, with no dramatic changes in G-values.

**Figure 3:**
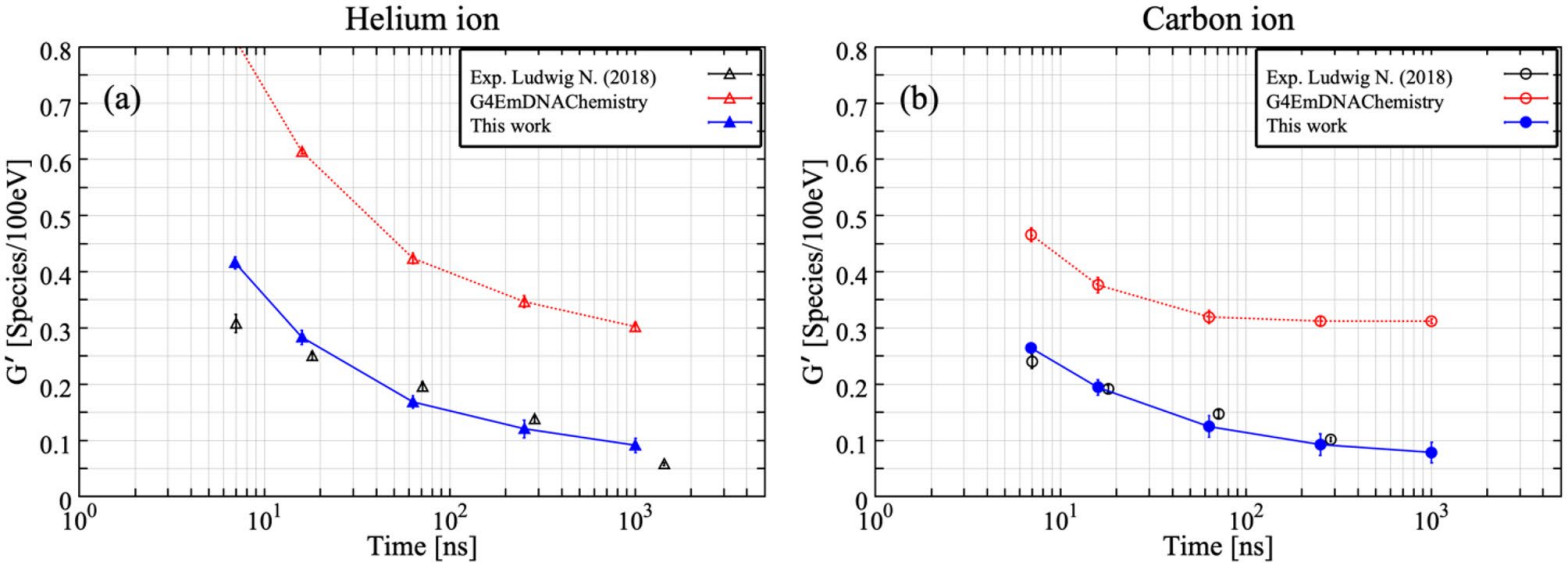
Time-dependent ^**·**^OH yields for (**a**) 0.75 MeV/u helium ions and (**b**) 0.83 MeV/u carbon ions. Experimental data presented by Ludwig N.^17^ were used for comparison with simulated time dependence of G-values.

**Figure 4:**
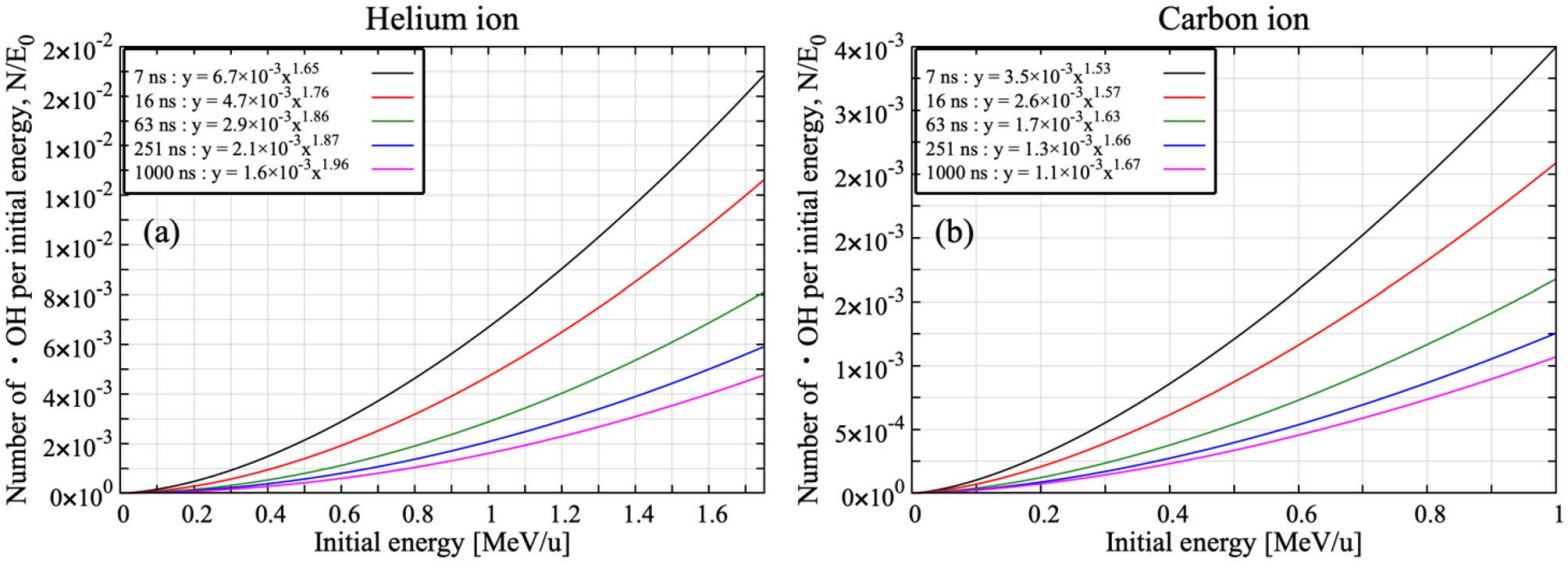
Number of ^**·**^OH produced per initial energy as a function of the initial energies of He (**a**) and C ions (**b**) 7, 16, 63, 251 and 1000 ns after irradiation.

**Figure 5:**
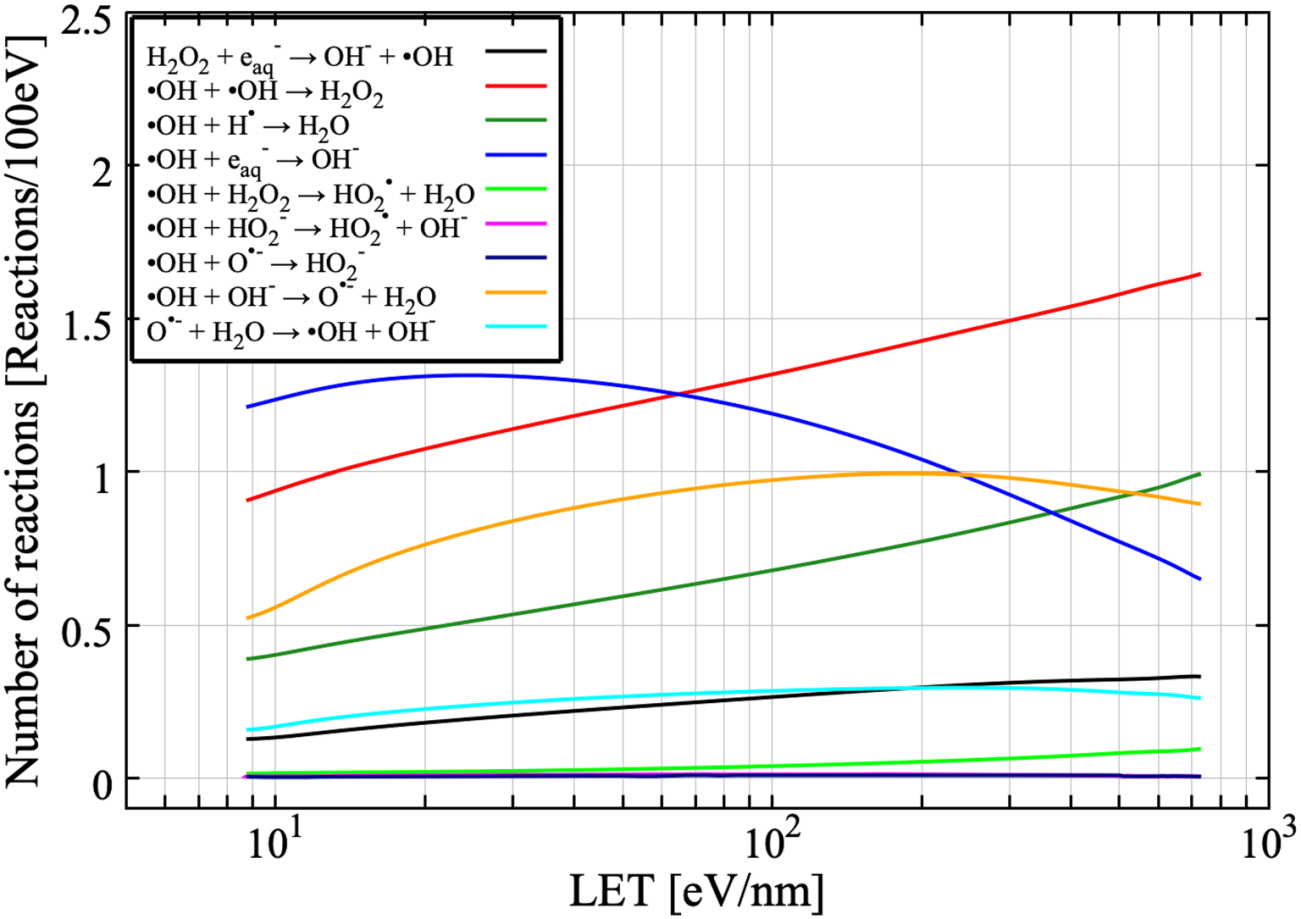
LET dependent number of reactions involving the ^**·**^OH at 1 μs after irradiation of a water target with C ions with a LET range of 10 eV/nm to 700 eV/nm. The number of reactions is the cumulative number of reactions up to 1 μs after irradiation.

When incoming ions (or electrons) have high enough energy, no drastic changes in G-value is visible (Fig. 2). But, to properly simulate the experimental results around Bragg peak energies^17^, at which incoming ions are completely stopped in the experimental set-up, we should consider the role played by secondary products added. Around the Bragg peak, many water radiolysis products would be generated along the ion trajectory due to high ionization density. Because of the high ionization density, it is necessary that radical–radical reactions are properly taken into account. Figure 3 shows the G-values of ^**·**^OH for 0.75 MeV/u He ions and 0.83 MeV/u C ions without the reaction of secondary products added (red: *G4EmDNAChemistry*). The experimental results are also plotted in black symbols^17^. The G-values of ^**·**^OH without the reaction of added secondary products are about twice higher than experimental results. Around the Bragg peak energy (< 6.0 MeV/u), high ionization density is expected compared to the high-energy region and incoming ions would completely stop in the irradiation cell. It is thus very tough to follow the reactions induced by incoming ions by a conventional CPU simulation. To more accurately simulate the G-value around the Bragg peak energy, we performed a split calculation. The number of ^**·**^OH produced was computed for each energy step with an increment of 0.25 MeV/u, and then, integrated from 0 to any energies as indicated in Fig. 4a,b. Finally, G-values are calculated from Eq. (2). All results shown in Fig. 4 have been calculated with the new reactions added. The time was chosen close to the scavenging ones of experimental data. When the role of reactions of added secondary products (Table 1) are taken into account, the discrepancy between simulation results (blue) and experimental results becomes significantly smaller than that without the reactions represented in Fig. 3. Figure 5 shows the number of reactions related to ^**·**^OH as a function of LET of C ions, ranging from 10 eV/nm to 700 eV/nm. The number of reactions is cumulative one up to 1 μs after the irradiation. Among newly added reactions, ^**·**^OH + OH^−^ → O^・−^ + H_2_O and O^・−^ + H_2_O → ^**·**^OH + OH^−^ contribute significantly to the reaction with ^**·**^OH. This means that we have to consider roles of newly added reactions to accurately simulate the G-values of water radiolysis products because these reactions contribute the annihilation process of ^**·**^OH.

### LET dependence of reactive species yield

**Figure 6:**
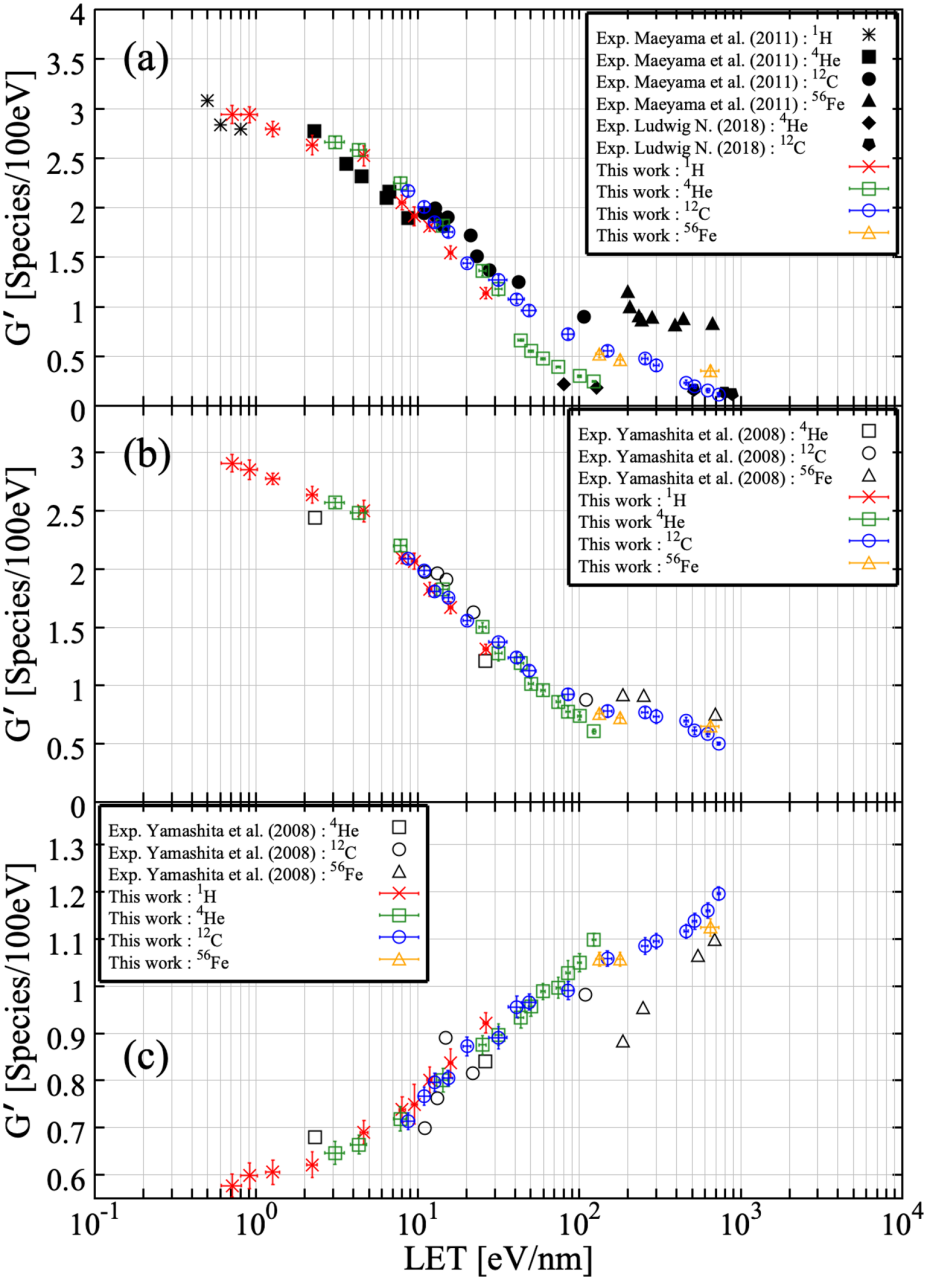
LET dependent G-values of (**a**) ^**·**^OH, (**b**) e_aq_^−^ and (**c**) H_2_O_2_ at 100 ns after irradiation of a water target with different radiation types. Sets of experimental data presented by Maeyama et al.^10^, Yamashita et al.^7^ and Ludwig^17^ were used for comparison with simulated LET dependence of G-values.

**Figure 7:**
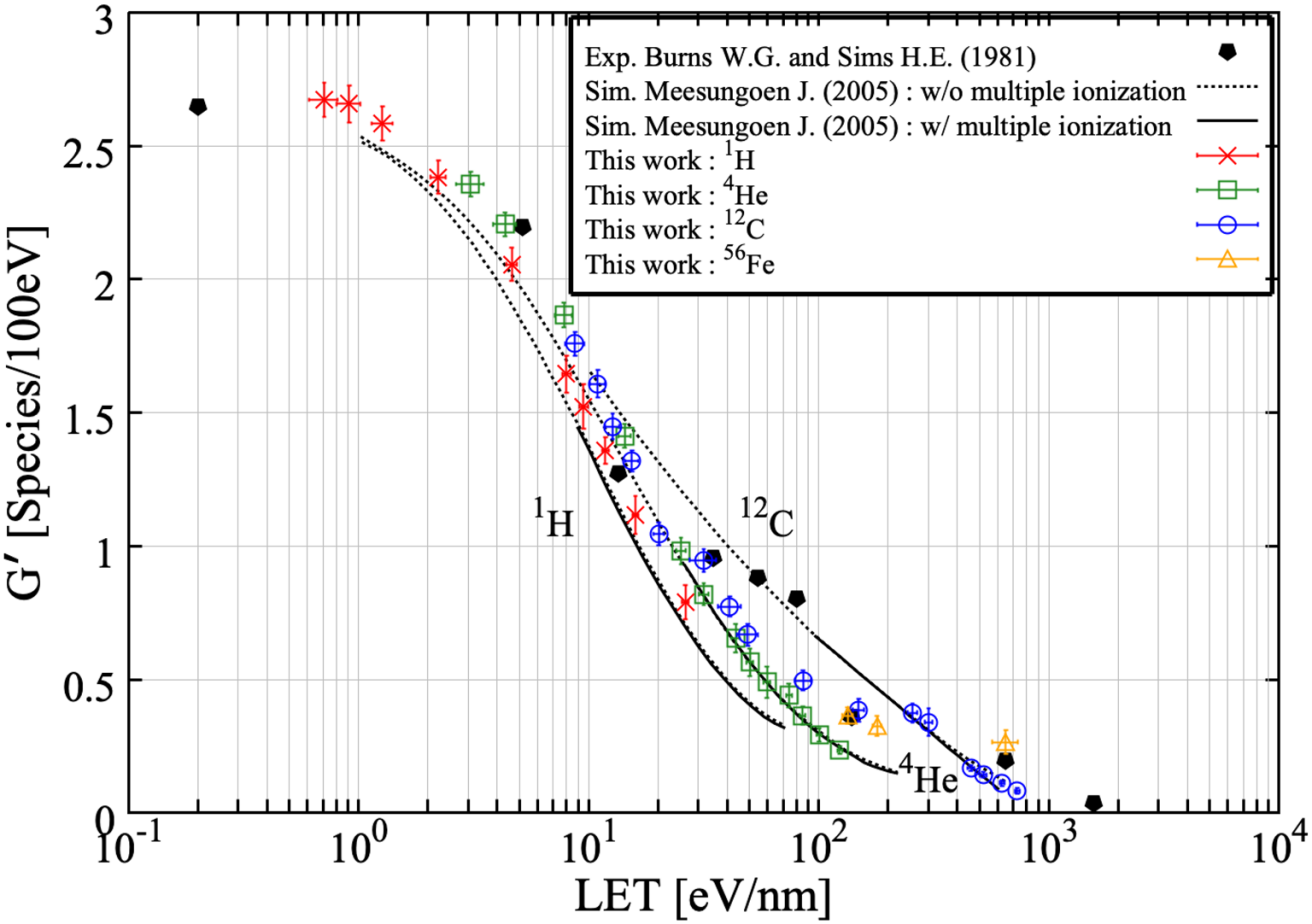
LET dependent G-values of ^**·**^OH at 1 μs after irradiation of a water target with different radiation types. The solid lines presented by Meesungunoen J. and Jay-Gerin J.P. represent the results of Monte Carlo simulations incorporating multi-ionization of water. The short-dot lines presented by by Meesungunoen J. and Jay-Gerin J.P. correspond to simulated G-values calculated as a function of LET without including the mechanism of multi-ionization of water^47^. Experimental data presented by Burns W.G. and Sims H.E.^50^ were used for comparison with simulated LET dependence of G-values.

Figure 6a–c show the LET dependence of G-values of ^**·**^OH, e_aq_^−^ and H_2_O_2_ at 100 ns after irradiation, simulated in this work and compared to experimental data. Overall, G-values of examined reactive species are in good agreement with experimental results (plotted points) in a wide LET range including the Bragg peak energies. In the cases of ^**·**^OH and e_aq_^−^, the G-value decreases monotonically with increasing LET as shown in Fig. 6a,b. In comparison to this, the G-value of H_2_O_2_ increases monotonically with increasing LET, with higher values for lighter ions at the same LET as shown in Fig. 6c. These results remind us that LET is not a universal parameter for describing the G-value. For scaling G-values, *Z*_*eff*_*/β* (or (*Z*_*eff*_*/β*)^2^), where *Z*_*eff*_ is the effective charge of incoming ions and *β* is the velocity of incoming ions normalized by the speed of light in vacuum, has been applied. Indeed, the description of the dependence of G-value is improved compared to that with LET, but *Z*_*eff*_*/β* has not been recognized as a universal parameter. To universally express G-values of water radiolysis products in a wide LET range, the electron interaction concept, which the number of interaction induced by secondary electrons governs the radiation induced yields could work well^7,44,45^. Furthermore, G-values of ^**·**^OH for Fe ions by the present simulation are significantly lower than the experimental results. In the experiments, a water equivalent moderator was used for adjusting the incident energy of Fe ions. Under this process, lighter ions, whose LET is smaller than that of Fe ions, could be produced by fragmentation. Considering the influence of lighter ions produced by fragmentation, higher G-values are expected, so it is reasonable that the present simulation result is lower than those of experiments. In the present version of Geant4-DNA, the influence of fragmentation of the ions is not considered. The implementation of the influence of the lighter ions produced by fragmentation would be one of the future issues to properly simulate the contribution of indirect action in the human body^46^. Moreover, discrepancies between simulations and experiments are confirmed in G-values of H_2_O_2._ The reason of this discrepancy is the implementation of multi-ionizations. In agreement with a previous study^47^, the influence of multi-ionization of air-free water molecules act effectively. Meesunguonen and Jay-Gerin suggested that the multi-ionizations, although less frequent compared to single ionization, especially contributes to the primary HO_2_^**·**^/O_2_^**·**−^ yield in the high LET region. In Meesungunoen J. and Jay-Gerin 's code named IONLYS-IRT, cross sections of elastic, phonon and vibrational electron scattering obtained from electron-impact on amorphous ice films have been employed. To take into account the effects of multi-ionization under high-LET heavy-ion irradiation the model has been extended to incorporate double, triple, and quadruple ionization process in single ion-water collisions. In the present simulation using Geant4-DNA, we consider the elastic scattering, ionization, electronic excitation and charge exchange processes for electron, proton and alpha particles. Furthermore, in the cases of heavy ions, only the ionization process is considered. In the absence of multi-ionization, the G-value of H_2_O_2_ increases monotonically with increasing LET as shown in Fig. 6c. When multi-ionizations are considered, we anticipate that the G-value of H_2_O_2_ would increase with increasing LET up to 100 eV/nm and then drop. Generally speaking, H_2_O_2_ is formed by the recombination of two ^**·**^OHs (^**·**^OH + ^**·**^OH → H_2_O_2_). As LET increases, radical–radical reactions occur more efficiently. Above 100 eV/nm, it is known that O(^3^*P*) is produced by multi-ionizations process. Since ^**·**^OH reacts not only with other ^**·**^OH but also with O(^3^*P*) (^**·**^OH + O(^3^*P*) → HO_2_^**·**^), ^**·**^OHs are consumed by other radical–radical reaction, causing a reduction of yields of H_2_O_2_ yield at high LET. However, we do not consider the contribution of the multi-ionization process in our simulation. Therefore, the roles of O(^3^*P*) are not added. H_2_O_2_ is formed not only by the recombination of two ^**·**^OHs, but also by the reaction between H^**·**^ and HO_2_^**·**^ (H^**·**^ + HO_2_^**·**^ → H_2_O_2_), and the reaction between H_3_O^+^ and HO_2_^−^ (H_3_O^+^  + HO_2_^−^ → H_2_O_2_). The previous simulation performed by Meesunguonen and Jay-Gerin more properly reproduced experimental G-values of H_2_O_2_ above 100 eV/nm. The current version of Geant4-DNA does not handle the multi-ionizations process. We are planning to add it into Geant4-DNA in the future. In comparison to e_aq_^−^, the influence of multi-ionizations to the G-value of ^**·**^OH is not significant. One of the main processes consuming the ^**·**^OH is its interaction with e_aq_^−^. This reaction is a competing process with the interaction of e_aq_^−^ with the O_2_^**·**−^. However, for the high LET region, ^**·**^OH react more efficiently with e_aq_^−^^48^. In brief, ^**·**^OH are diminished by reactions with e_aq_^−^ in the track core. This explains why the present simulation results of ^**·**^OH are in good agreement with experimental results as represented in Fig. 6a. In accordance with previous studies, ^**·**^OH and e_aq_^−^ are not affected by the multi-ionization^47,49^. Furthermore, ^**·**^OH react with e_aq_^−^ in the high LET region, dense track on the particle path^47,49^. Thus, although multi-ionization processes are not considered in the simulation, the present results regarding ^**·**^OH and e_aq_^−^ are in good agreement with experimental results. Figure 7 shows a comparison of the simulated G-values of ^**·**^OH at 1 μs after irradiation to a previous simulation^47^ and experimental measurements^50^. The present simulations (open symbols) are in good agreement with the previous simulations (solid lines) and experimental results (solid symbols). Once again, the present simulations successfully reproduced the experimental results in a wide LET range thanks to the consideration of newly added reactions.

## Conclusions

In the present study, we simulated the track segment yields (G′) of water radiolysis products (^**·**^OH, e_aq_^−^ and H_2_O_2_) under heavy ion irradiation using a Monte Carlo simulation code in the Geant4-DNA in a wide LET range up to 700 eV/nm. To accurately simulate the G′ around the Bragg peak, we addressed two issues. The first issue is roles played by secondary products generated by water radiolysis. The second issue is the long computation time due to the abundantly generated water radiolysis products around the Bragg peak. To save the computation time, we did the split simulation. Consequently, G′ of ^**·**^OH and e_aq_^−^ were in good agreement with experimental results in the examined LET range. However, discrepancies of G′ of ^**·**^OH between simulation and experiment were seen in Fe ions. This discrepancy suggested that the contribution of lighter particles produced by fragmentation should be considered for a more accurate simulation. At the same LET, G’ of ^**·**^OH and e_aq_^−^ of heavier ions were lower than that of lighter one, especially noticeable from 40 to 100 eV/nm. This finding is consistent with the fact that LET is not universal parameter to express the yields of water radiolysis products especially when it come to complex track structure with high energy secondary electron. At this stage *Z*_*eff*_*/β* could be a better parameter. It will be crucial to discuss a parameter that universally describe the G′ of water radiolysis products. In comparison to ^**·**^OH and e_aq_^−^, G′ of H_2_O_2_ did not agree with experimental results above 100 eV/nm even after the consideration of roles of secondary products generated by water radiolysis. This is because the contribution of multi-ionization is not taken into account in the present stage. To have a more realistic simulation, the multi-ionization should be added in the Geant4-DNA in the near future.
